# Monitoring and Measurement in Child and Adolescent Mental Health: It’s about More than Just Symptoms

**DOI:** 10.3390/ijerph19084616

**Published:** 2022-04-12

**Authors:** Jenna Jacob, Julian Edbrooke-Childs

**Affiliations:** 1 CORC, Applied Research and Evaluation, Anna Freud Centre, 4–8 Rodney Street, London N1 9JH, UK; julian.childs@annafreud.org; 2 Clinical, Education and Health Psychology, UCL, Gower Street, London WC1E 6AE, UK

Routine outcome monitoring (ROM) provides information to practitioners and others providing healthcare support to demonstrate the impact of interventions and for service evaluation. ROM usually takes the form of questionnaires that are regularly administered to patients, from which healthcare professionals can gauge to what extent therapy is progressing [1]. Such questionnaires can be used as clinical tools that may help facilitate better collaborative practice; to extrapolate discussions about how a young person or family sees progress and their experiences of the work with the practitioner [2]. ROM is widely used in youth mental health services [2,3,4,5] although not without its challenges, including limited resources, and lack of clinical “buy-in” [3,6,7,8,9]. The majority of ROM questionnaires are standardized, comprising fixed items that span difficulties identified by clinicians most often focused on symptomology [10] and often specific to certain population groups, e.g., age range; learning disabilities; perinatal.

Evidence suggests that clinicians are less accurately able to predict patient outcomes using clinical judgement alone, particularly when a patient is veering off a projected track of progress [11,12]. It is also important to consider the differences between the use of measures and the use of measures with feedback: of incorporating discussion about the measurement questions, and areas of tracking focus, into the work with young people, and families/carers, as relevant. The importance of using outcome measures in a considered way is a matter of ethical concern and has been advised for a number of years [2]. This is where the use of measures and the collection of data are conducted in a way that is meaningful to the client and practitioner, and is not just a bureaucratic exercise, absent of any discussion with the client. Further, central to this considered use of ROM is discussion and focus on areas of outcome tracking that are important to the young person [10,13,14,15]. When used as part of collaborative practice, shared decision-making, and to empower clients, the use of ROM has been perceived by clients as helpful, as a means to enhance communication and to stimulate reflection [2,16]. Goal setting as ROM has been described as a conduit for a shared understanding of difficulties and ways forward [17,18], and key to young people feeling heard, valued and understood [2,18,19].

In a review of evidence regarding the impact of feedback from outcome measures on treatment effectiveness, incorporating feedback from standardized measures into ongoing therapy sessions with youth has been evidenced as useful in just over half of all cases [20]. Of particular importance was the use of feedback to ensure better outcomes for those who are veering off a projected trajectory, and better outcomes are reported when feedback is provided to both the practitioner and the patient [20]. In a meta-analysis from around the same time, Tam and Ronan [21] found a small but significant effect size of 0.20 (Hedges’s g) for feedback with youth. However, the authors of both studies advise caution in interpretation due the considerable heterogeneity across the reviewed studies and outcome measures. As far as we are aware, there have been no more recent reviews of the literature to further these findings, and recent Cochrane reviews highlight a paucity of evidence generally in both adult and youth settings [22,23]. Research to date has prioritized the experiences of youth in the use of routine outcome measures, which is of paramount importance.

There are several examples of measurement-focused care worldwide e.g., [2,24,25,26]. However, these initiatives tend to focus on symptomology tracking, as opposed to more complementary elements of change and improvement, such as functioning or empowerment. Whilst a focus on symptoms can be important, it will not be the principle area of change for all. The challenges of relying heavily on symptomology are that other important areas of outcome may be missed. For example, evidence demonstrates that widely available youth outcome measures do not capture the gamut of goals set by youth for focus in therapy, including existential areas of improvement such as understanding oneself, being independent or responsible, and increasing confidence [14]. A recent systematic review also reported that clients of all ages can experience ROM as suspicious, and limited in its ability to fully capture their needs [16]. Further, focusing solely on only one area of change, namely symptomology, runs the risk of over- or under- reporting levels of change; varied levels of improvement have been demonstrated when focused on different areas of measurement. Evidence suggests that only around 16% of youths report consistent cross-domain improvement, and one in four young people have reported significant improvements in symptoms but not in functioning. Further, one in three young people reported significant goal progress but not significant symptom improvement [27], and when goal data were added to combined analysis of symptom-based outcome analysis, an increase in the overall level of measurable improvement and a reduction in the no measurable change category was reported for youths [28]. 

There are also issues with the inclusiveness of self-report measures in particular. As text-based methods, these outcome measures could exclude some young people, either due to cultural or linguistic reasons, or because they have learning disabilities. Such examples include the propensity for standardized outcome measures to be developed based on experiences, behaviors, and attitudes of young White people from the Global North, which is underpinned by a general lack of research focused on young people in the Global South [29], despite 85% of the world’s youth being in Global South countries [30]. Further, mental health and wellbeing measure questions have been found to not be age-appropriate [31], and young people have raised challenges associated with the readability of them [32], also highlighting the frequent disparities between chronological age and reading age. These factors render a mismatch between standardized questions and many young people’s understanding and interpretation of their own experiences, as well as the understanding and interpretation of the data derived from the measures. The measurement of patients who have experienced trauma is also often excluded from exploration (e.g., [33]), or not adequately capturing relevant areas of outcome [34]. This further highlights the importance of considering complementary areas and methods of ROM.

This Special Issue is focused on a wide range of ROM. This includes measurement considerations for tracking quality of life (Krause et al.) and empowerment (Harju-Seppänen et al.), contextualizing, and complexity factors (da Costa et al.), the exploration of goal setting for use with young people who have experienced trauma (Law et al.), and who are receiving mental health interventions in school (Duncan et al.), the exploration of mental and physical health outcomes for children and young people with chronic physical health difficulties (Hiremath et al.), and for those who use the dark web (Idelji-Tehrani et al.). Tying all of these studies together, the clear need for additional monitoring outside of traditional symptom tracking is required for youth mental health and wellbeing support. It has been long recommended that individualized measures, or those that seek to explore a holistic view of outcomes, are used alongside standardized symptom focused outcome measures to ensure a breadth of information is explored [35,36,37,38]. By reflecting on the monitoring and measurement in child and adolescent mental health through this wide range of methods and approaches, we hope that this Special Issue furthers learning in the field. Through this multifaceted exploration of ROM, we hope it helps to promote reflection, which is useful at all levels of service provision, from the use of measures by practitioners, to wider policy and research considerations, retaining at the heart a considered focus on young people as whole beings and with areas of important outcome measurement which reach beyond a sole focus on symptoms.
